# Quality of Life Among Attendants/Caregivers of Dialysis Patients

**DOI:** 10.7759/cureus.83989

**Published:** 2025-05-12

**Authors:** Aurangzeb Afzal, Hafza Sumaira Rahman, Muhammad Ahmad Rauf, Zahid Rafique, Areeba Gulzar, Waqas Rasheed, Jehangir Afzal Mobushar, Beenish Abbas Bajwa, Iqra Naeem, Muhammad Irfan Jamil

**Affiliations:** 1 Nephrology, Services Hospital Lahore, Lahore, PAK; 2 Medicine, Rashid Hospital, Dubai, ARE; 3 Internal Medicine, Services Hospital Lahore, Lahore, PAK; 4 Research, Services Hospital Lahore, Lahore, PAK; 5 Psychiatry and Behavioral Sciences, Lahore General Hospital, Lahore, PAK; 6 Nephrology, Lahore General Hospital, Lahore, PAK

**Keywords:** caregivers, dialysis, end-stage renal disease (esrd), hemodialysis related, maintenance hemodialysis, quality of life of caregiver

## Abstract

Aim and background: Caregivers of dialysis patients often face substantial physical, psychological, and social challenges impacting their quality of life. This study aimed to evaluate caregivers' quality of life across multiple domains using the World Health Organization Quality of Life-BREF (WHOQOL-BREF) assessment tool.

Methods: A hospital-based cross-sectional study was conducted at the Dialysis Center of Services Hospital Lahore from March to August 2024. A total of 164 caregivers of adult patients undergoing maintenance hemodialysis were enrolled using consecutive sampling after obtaining written informed consent. Data were collected through face-to-face interviews using a structured Urdu-language questionnaire incorporating the WHOQOL-BREF tool. Caregivers aged ≥18 years, providing unpaid care for ≥3 months, were included. WHOQOL-BREF assessed four quality-of-life domains. Data were analyzed using IBM SPSS Statistics for Windows, Version 26 (Released 2019; IBM Corp., Armonk, New York), with p ≤ 0.05 considered statistically significant.

Results: Out of 164 caregivers enrolled, 88 (53.7%) were male and 76 (46.3%) female, with the majority being married and having at least intermediate education. Most caregivers were sons, wives, or daughters of the dialysis patients. The mean scores across WHOQOL-BREF domains were physical health 50.01 ± 13.70, psychological health 54.10 ± 14.50, social relationships 60.83 ± 22.08, and environmental domain 51.57 ± 16.91. No statistically significant associations were observed between physical, psychological, or environmental domain scores and gender, marital status, education level, age group, or caregiver-patient relationship (p > 0.05). However, the social relationships domain showed a statistically significant difference across age groups (p = 0.017), with higher scores in older caregivers. All other domain comparisons remained statistically non-significant (p > 0.05). Overall, caregiver quality of life was highest in the social domain and lowest in the physical domain.

Conclusion: Caregivers of dialysis patients reported moderate overall quality of life, with the highest scores in social relationships and the lowest in physical health. While caregiver age significantly influenced social domain scores, other sociodemographic factors (gender, marital status, education, and relationship) did not show significant associations. These findings highlight the need for targeted interventions addressing physical health and age-specific social support.

## Introduction

Chronic kidney disease (CKD) is a growing global health concern, and many patients progress to end-stage renal disease (ESRD), requiring maintenance dialysis for survival. Hemodialysis, the most widely utilized renal replacement therapy for ESRD, can sustain life but also imposes major disruptions on patients' daily routines and functional status [1]. Frequent dialysis sessions, strict dietary and fluid restrictions, and treatment-related fatigue significantly restrict patients' ability to work and perform daily activities, which in turn impacts not only patients but also the family members or caregivers [2-4].

Caregivers play a crucial role in managing chronic illnesses like ESRD by providing physical assistance, emotional support, and coordination of care for dialysis patients. These caregivers are often spouses, children, or other relatives who receive no formal compensation yet spend considerable time and energy to help patients adhere to treatment and adapt to lifestyle changes [5,6]. However, the continuous demands of dialysis care place caregivers under substantial stress; they frequently experience emotional strain, physical exhaustion, social isolation, and financial difficulties as a direct result of their caregiving responsibilities. Indeed, caregivers of hemodialysis patients are sometimes termed "hidden patients," given their heightened risk of developing various physical and mental health problems, which often go unrecognized [4,6].

Studies have shown that family caregivers of dialysis patients often report lower quality of life (QoL) compared to age-matched individuals in the general population. Key domains such as psychological well-being and social relationships tend to be most compromised; caregivers carry a significant burden in maintaining the patient's well-being and face an elevated risk of depression, particularly when social support is inadequate [7,8]. A systematic review noted that although caregivers' overall QoL may be somewhat better than that of the patients they care for, it remains substantially diminished relative to healthy individuals [9].

Although caregiver burden in dialysis is globally acknowledged, local data from Pakistan remain scarce. Cultural expectations, joint family systems, and limited resources likely shape caregiving experiences differently. However, the specific QoL domains affected in caregivers here are not well documented. A structured evaluation is essential to identify challenges and improve caregiver support. Therefore, this study aimed to assess the QoL of caregivers of hemodialysis patients at a tertiary care hospital using the World Health Organization Quality of Life-BREF (WHOQOL-BREF) tool, where "BREF" denotes the abbreviated version of the WHOQOL-100. It includes 26 items covering four domains: physical health, psychological health, social relationships, and environmental factors. This validated instrument has been widely applied in both patient and caregiver populations, including caregivers of individuals undergoing maintenance hemodialysis, due to its cross-cultural adaptability, simplicity, and psychometric strength.

## Materials and methods

This hospital-based cross-sectional study was conducted at the Dialysis Center of Services Hospital Lahore, a tertiary care teaching hospital in Pakistan. The study was carried out between March and August 2024 after obtaining written informed consent from all participants. Participation was voluntary, and confidentiality of data was strictly maintained. Ethical approval was taken from the Institutional Review Board Services Institute of Medical Sciences/Services Hospital Lahore (IRB/2023/1126/SIMS). The study population consisted of caregivers of adult patients undergoing maintenance hemodialysis. A caregiver was defined as a family member or unpaid attendant primarily responsible for assisting a dialysis patient with daily activities and hospital visits. Inclusion criteria were caregivers aged ≥18 years who had been caring for a patient on maintenance hemodialysis for at least three months. A sample size of 173 was calculated using a mean difference of 0.6 in WHOQOL-BREF scores (SD = 1.63) based on prior research with a 95% confidence interval, 5% level of significance, and a 10% adjustment for non-response; using consecutive sampling, a total of 164 eligible caregivers who provided informed consent were ultimately enrolled [10]. Data were collected through face-to-face interviews using a questionnaire. The questionnaire included items on caregiver demographics (age, sex, education, employment, relationship to patient, caregiving duration) and incorporated the WHOQOL-BREF tool. Interviews were conducted in the Urdu language to ensure clarity and comprehension. Education level was categorized based on completed school years as follows: no formal education (0 years), primary (Grades 1-5), middle (Grades 6-8), matriculation (Grades 9-10), intermediate (Grades 11-12), and graduation or higher (Grade 13 and above).

Caregivers' QoL was measured using the WHOQOL-BREF questionnaire. The WHOQOL-BREF consists of 26 items covering four QoL domains: Physical Health, Psychological Health, Social Relationships, and Environment, as well as two general items for overall QoL and general health. Each item is rated on a five-point Likert scale, with domain scores transformed to a 0-100 range, where higher scores indicate better QoL. Renowned for its reliability and validity, the WHOQOL-BREF is suitable for both clinical and large-scale research, and its availability in multiple languages enhances its utility in cross-cultural studies [11]. As the questionnaire was completed in one sitting with researcher facilitation, there were no missing data. All responses were checked for completeness at the time of the interview.

Data were analyzed using IBM SPSS Statistics for Windows, Version 26 (Released 2019; IBM Corp., Armonk, New York). Descriptive statistics (mean ± standard deviation for continuous variables; frequencies and percentages for categorical variables) were used to summarize caregiver characteristics and WHOQOL-BREF domain scores. Caregiver's characteristics were stratified, and post-stratification analysis was conducted using appropriate tests (independent t-tests or one-way ANOVA). A p-value < 0.05 was considered statistically significant for all analyses.

## Results

Out of 164 caregivers included in the study, 88 (53.7%) were males, and 76 (46.3%) were females. Regarding education levels, 13 (7.9%) caregivers had no formal education, 21 (12.8%) were educated up to the primary level, 19 (11.6%) had education up to the middle level, 24 (14.6%) had completed matriculation, 45 (27.4%) had intermediate education, and 42 (25.6%) had graduation or higher qualifications. Marital status indicated that 63 (38.4%) caregivers were single, whereas 101 (61.6%) were married. The caregivers' relationships to patients included brothers 25 (15.2%), sisters 15 (9.1%), husbands 19 (11.6%), wives 37 (22.6%), sons 45 (27.4%), and daughters 23 (14.0%).

The mean QoL scores among caregivers of dialysis patients across the four WHOQOL-BREF domains were as follows: physical health 50.01 ± 13.70, psychological health 54.10 ± 14.50, social relationships 60.83 ± 22.08, and environmental domain 51.57 ± 16.91. Overall, the highest mean QoL was observed in the social relationships domain, while the lowest was in the physical health domain.

No statistically significant differences were observed in physical health domain scores across gender (p = 0.851), marital status (p = 0.291), education level (p = 0.711), age groups (p = 0.056), or relationship to the patient (p = 0.461). Age groups showed an increasing trend in mean scores, but the difference remained non-significant (Table 1).

**Table 1:** Comparison of Physical Health Domain Scores Across Sociodemographic Variables. Physical health domain scores across caregiver variables using t-tests (gender, marital status) and ANOVA (age, education, relationship); df indicates between-group degrees of freedom. A p-value of ≤ 0.05 is considered statistically significant. SD = standard deviation, CI = confidence interval, df = degrees of freedom.

Variable	Group (n)	Mean ± SD	95% CI	Test Value	p-value
Gender	Male (88)	49.82 ± 14.35	-4.65–3.84	-0.188	0.851
	Female (76)	50.22 ± 12.99			
Marital Status	Single (63)	48.57 ± 14.39	-6.67–2.01	-1.059	0.291
	Married (101)	50.90 ± 13.25			
Age Group (Years)	18–30 years (102)	48.25 ± 14.54	45.39–51.10	2.942	0.056
	31–45 years (45)	51.69 ± 11.07	48.36–55.02		
	Above 45 years (17)	56.12 ± 13.19	49.34–62.90		
Education Level	No formal education (13)	50.23 ± 13.24	42.23–58.23	0.585	0.711
	Up to primary (21)	52.24 ± 15.12	45.35–59.12		
	Middle (19)	48.84 ± 11.73	43.19–54.50		
	Matriculation (24)	52.71 ± 13.94	46.82–58.59		
	Intermediate (45)	47.67 ± 14.93	43.18–52.15		
	Graduation and above (42)	50.31 ± 12.70	46.35–54.27		
Relationship	Brother (25)	53.96 ± 13.07	48.57–59.35	0.934	0.461
	Sister (12)	48.80 ± 15.27	40.35–57.25		
	Husband (19)	50.89 ± 11.95	45.13–56.66		
	Wife (37)	51.51 ± 11.56	47.66–55.37		
	Son (45)	47.36 ± 15.60	42.67–52.04		
	Daughter (23)	48.52 ± 13.93	42.50–54.54		

Psychological health domain scores showed no statistically significant differences across gender (p = 0.207), marital status (p = 0.398), education level (p = 0.178), age groups (p = 0.098), or relationship to the patient (p = 0.084). Although higher mean scores were noted with increasing age and among sisters and graduates, all comparisons remained non-significant (Table 2).

**Table 2:** Comparison of Psychological Health Domain Scores Across Sociodemographic Variables. Psychological health domain scores across caregiver variables using t-tests (gender, marital status) and ANOVA (age, education, relationship); df indicates between-group degrees of freedom. A p-value ≤ 0.05 is considered statistically significant. SD = standard deviation, CI = confidence interval, df = degrees of freedom.

Variable	Group (n)	Mean ± SD	95% CI	Test Value	p-value
Gender	Male (88)	52.77 ± 15.27	-7.35–1.60	-1.267	0.207
	Female (76)	55.64 ± 13.48			
Marital Status	Single (63)	52.89 ± 16.07	-6.57–2.63	-0.847	0.398
	Married (101)	54.86 ± 13.45			
Age Group (Years)	18–30 years (102)	52.74 ± 15.45	49.70–55.77	2.352	0.098
	31–45 years (45)	54.67 ± 12.76	50.83–58.50		
	Above 45 years (17)	60.82 ± 11.22	55.05–66.59		
Education Level	No formal education (13)	48.15 ± 16.73	38.04–58.27	1.549	0.178
	Up to primary (21)	56.05 ± 15.06	49.19–62.90		
	Middle (19)	55.37 ± 12.84	49.18–61.56		
	Matriculation (24)	56.42 ± 14.82	50.16–62.67		
	Intermediate (45)	50.53 ± 15.82	45.78–55.29		
	Graduation and above (42)	56.90 ± 11.83	53.22–60.59		
Relationship	Brother (25)	56.04 ± 12.49	50.89–61.19	1.984	0.084
	Sister (12)	59.67 ± 15.02	51.35–67.98		
	Husband (19)	57.00 ± 13.35	50.57–63.43		
	Wife (37)	56.03 ± 13.12	51.65–60.40		
	Son (45)	49.11 ± 16.61	44.12–54.10		
	Daughter (23)	52.65 ± 13.13	46.97–58.33		

The social relationships domain scores showed no statistically significant differences across gender (p = 0.616), marital status (p = 0.154), education level (p = 0.867), or relationship to the patient (p = 0.384). However, a statistically significant difference was found across age groups (p = 0.017), with scores increasing progressively from younger to older caregivers (Table 3).

**Table 3:** Comparison of Social Relationships Domain Scores Across Sociodemographic Variables. The table presents social relationships domain scores across caregiver variables using t-tests (gender, marital status) and ANOVA (age, education, relationship); df indicates between-group degrees of freedom. A p-value ≤ 0.05 is considered statistically significant. SD = standard deviation, CI = confidence interval, df = degrees of freedom.

Variable	Group (n)	Mean ± SD	95% CI	Test Value	p-value
Gender	Male (88)	60.02 ± 25.24	-8.58–5.10	-0.502	0.616
	Female (76)	61.76 ± 17.86			
Marital Status	Single (63)	57.71 ± 22.38	-12.04–1.92	-1.431	0.154
	Married (101)	62.77 ± 21.78			
Age Group (Years)	18–30 years (102)	57.16 ± 22.00	52.84–61.48	4.158	0.017
	31–45 years (45)	65.60 ± 20.06	59.57–71.63		
	Above 45 years (17)	70.24 ± 23.73	58.03–82.44		
Education Level	No formal education (13)	56.23 ± 29.08	38.66–73.80	0.373	0.867
	Up to primary (21)	61.05 ± 20.55	51.69–70.40		
	Middle (19)	60.53 ± 13.70	53.92–67.13		
	Matriculation (24)	62.21 ± 25.65	51.38–73.04		
	Intermediate (45)	58.62 ± 23.15	51.67–65.58		
	Graduation and above (42)	63.86 ± 20.87	57.35–70.36		
Relationship	Brother (25)	60.52 ± 24.57	50.38–70.66	1.061	0.384
	Sister (12)	68.33 ± 20.69	56.88–79.79		
	Husband (19)	67.84 ± 24.13	56.21–79.47		
	Wife (37)	60.49 ± 18.37	54.36–66.61		
	Son (45)	56.91 ± 25.81	49.16–64.66		
	Daughter (23)	58.70 ± 14.30	52.51–64.88		

Environmental domain scores showed no statistically significant differences across gender (p = 0.927), marital status (p = 0.360), education level (p = 0.627), age groups (p = 0.061), or relationship to patient (p = 0.858). Although mean scores increased with age and education, none of the observed differences reached statistical significance (Table 4).

**Table 4:** Comparison of Environmental Domain Scores Across Sociodemographic Variables. The table presents social relationships domain scores across caregiver variables using t-tests (gender, marital status) and ANOVA (age, education, relationship); df indicates between-group degrees of freedom. A p-value ≤ 0.05 is considered statistically significant. SD = standard deviation, CI = confidence interval.

Variable	Group (n)	Mean ± SD	95% CI	Test Value	p-value
Gender	Male (88)	51.45 ± 17.30	-5.49–5.00	-0.091	0.927
	Female (76)	51.70 ± 16.55			
Marital Status	Single (63)	50.03 ± 17.57	-7.86–2.87	-0.918	0.360
	Married (101)	52.52 ± 16.50			
Age Group (Years)	18–30 years (102)	49.62 ± 17.19	46.24–52.99	2.853	0.061
	31–45 years (45)	52.91 ± 16.05	48.09–57.73		
	Above 45 years (17)	59.71 ± 15.50	51.74–67.67		
Education Level	No formal education (13)	50.62 ± 16.97	40.36–60.87	0.697	0.627
	Up to primary (21)	52.10 ± 20.73	42.66–61.53		
	Middle (19)	48.16 ± 16.19	40.35–55.96		
	Matriculation (24)	52.29 ± 17.22	45.02–59.56		
	Intermediate (45)	49.33 ± 14.60	44.95–53.72		
	Graduation and above (42)	55.12 ± 17.54	49.65–60.58		
Relationship	Brother (25)	53.36 ± 13.95	47.60–59.12	0.386	0.858
	Sister (12)	53.40 ± 20.57	42.01–64.79		
	Husband (19)	54.42 ± 16.37	46.53–62.31		
	Wife (37)	51.84 ± 13.58	47.31–56.37		
	Son (45)	49.53 ± 19.32	43.73–55.34		
	Daughter (23)	49.61 ± 18.52	41.60 to 57.62		

## Discussion

This cross-sectional study assessed QoL among hemodialysis caregivers in Pakistan using WHOQOL-BREF. The mean domain scores were 50.01 (physical), 54.10 (psychological), 60.83 (social), and 51.57 (environment). Caregivers showed moderate overall QoL, with greater challenges in physical and environmental domains. No significant differences were found across gender, marital status, education, or caregiver relationship. However, older caregivers reported significantly higher social domain scores (p = 0.017), suggesting age-related variation in perceived social support.

The mean physical health score of 50 reflects notable physical strain among caregivers. This is lower than scores reported in South India (68%) [12] and Brazil (70%) [13], suggesting a greater burden in our setting. Caregiving duties such as assisting with mobility, managing dialysis sessions, and prolonged hospital visits may contribute to fatigue and neglected personal health. These factors often lead to sleep issues and untreated illnesses. Previous studies also highlight the physical risks caregivers face [14]. Our findings reinforce the physical toll of caregiving, which may have a more severe impact on this population.

The mean psychological domain score reflected moderate distress among caregivers, though not the lowest among QoL domains. Global data often highlight psychological strain as a key issue; one review noted greater disturbance in the mental aspects of QoL [9]. This study's score, while slightly higher, still suggests stress, anxiety, or low mood. Cultural factors such as religious belief, family support, and a sense of duty may help Pakistani caregivers cope, offering some resilience against mental health challenges [9,15]. The lack of a gender difference in psychological scores is notable. Women in caregiving roles often report higher stress than men [9], yet no such disparity was found here. Male caregivers may experience similar strain, or cultural norms may limit the expression of distress. The psychological toll remains significant for all caregivers. Another Pakistani study reported an inverse association between caregiver burden and QoL [16], with higher burden linked to poorer mental health [17].

The social relationships domain was the highest-scoring domain (~60.8), indicating preserved interpersonal support. This aligns with Pakistan's extended family system, where caregivers often receive emotional or practical help. Comparable data also show relatively better social QoL in other populations, such as Indian caregivers (13.9/20, ~69%) [17] and Brazilian caregivers (10.7/15, ~71%) [13]. Older caregivers scored higher in social relationships, possibly due to fewer obligations, greater acceptance, and stronger networks. Younger adults often juggle work and family, risking social isolation. A review noted that younger caregivers had lower QoL [9]. Another study reported an increasing burden with advancing age [14].

The environmental domain of QoL was also notably low in our study (mean ~51.6/100), on par with the physical domain in its level of impairment. The environment domain encompasses financial resources, healthcare access, safety, and living conditions. A score around 50 indicates that caregivers are dissatisfied with many environmental aspects. This is not surprising, as chronic dialysis imposes financial strain and lifestyle disruptions on families. Many caregivers in Pakistan bear significant out-of-pocket costs for travel to dialysis centers, medications, or special diets for patients, which can cause economic hardship. Time spent caregiving may reduce their income if they have to cut back work hours or leave jobs. Furthermore, the hospital-centered nature of thrice-weekly dialysis means caregivers spend a lot of time in healthcare settings that may be crowded or far from home, affecting their comfort and convenience. When comparing internationally, our caregivers' environment scores appear somewhat lower than those documented in higher-income settings. For instance, caregivers in another study had an environment mean score of 25.2 (raw) [13], approximately 63% of the maximum, whereas our mean corresponds to ~52%. Even in the Indian study, the environment domain mean was 12.67 out of 20 (~63% on a 0-100 scale) [17], higher than our findings.

Gender, marital status, education, and caregiver-patient relationship did not significantly influence QoL, implying a universal caregiving effect. Some research shows that female caregivers have worse QoL [9,13,14] due to heavier tasks, though men may receive female relatives' support [18-20]. Education also failed to protect against reduced QoL, aligning with evidence that high caregiving burden negates any advantage [9,13,17,20]. Other studies suggest that higher education aids in accessing information, but caregiving stress likely overrides these benefits. Regression analysis showed no significant sociodemographic predictors, except age on social QoL, suggesting minimal influence or potential importance of unmeasured factors (e.g., caregiving hours, patient severity, coping style). A recent local study similarly found no demographic effects on QoL when burden and stress were accounted for, confirming these results [16].

Several potential biases and limitations exist. This cross-sectional design only captures QoL at one point, limiting causal inference. Certain resilient individuals may self-select as caregivers, complicating directionality. Sampling from a single hospital might exclude more stressed caregivers, underestimating the true burden, while a "healthy caregiver" effect is also possible. Social scores may be inflated by hospital-based interactions. Self-reporting can cause underreporting bias. A moderate sample size and uneven subgroups reduce power to detect differences. Caregiving duration and weekly hours, known influences on stress, were unaccounted for, leading to an incomplete picture. Overall, the study underestimates caregiving burden, indicating greater challenges.

## Conclusions

The study found that caregivers of dialysis patients experienced moderate QoL across physical, psychological, social, and environmental domains, with notably lower physical health scores and relatively better social relationships. Among the sociodemographic variables analyzed, only caregiver age showed a statistically significant association with the social relationships domain, demonstrating improved scores among older caregivers. However, gender, marital status, education level, and caregiver-patient relationship did not significantly influence QoL scores in any domain. These results suggest the importance of designing comprehensive support programs focusing on physical well-being and considering age-specific strategies to enhance social support for younger caregivers.
